# Exploring the Intersection of Geophysics and Diagnostic Imaging in the Health Sciences

**DOI:** 10.3390/diagnostics14020139

**Published:** 2024-01-08

**Authors:** Rahul Kumar Singh, Nirlipta Priyadarshini Nayak, Tapan Behl, Rashmi Arora, Md. Khalid Anwer, Monica Gulati, Simona Gabriela Bungau, Mihaela Cristina Brisc

**Affiliations:** 1 Energy Cluster, University of Petroleum and Energy Studies, Dehradun 248007, Uttarakhand, India; rahulssgk3806@gmail.com (R.K.S.); npnayak@ddn.upes.ac.in (N.P.N.); 2 Amity School of Pharmaceutical Sciences, Amity University, Mohali 140306, Punjab, India; 3 Chitkara College of Pharmacy, Chitkara University, Rajpura 140401, Punjab, India; rashmi.arora@chitkara.edu.in; 4 Department of Pharmaceutics, College of Pharmacy, Prince Sattam Bin Abdulaziz University, Alkharj 11942, Saudi Arabia; m.anwer@psau.edu.sa; 5 School of Pharmaceutical Sciences, Lovely Professional University, Phagwara 1444411, Punjab, India; monicagulati14@gmail.com; 6 Australian Research Centre in Complementary and Integrative Medicine, Faculty of Health, University of Technology Sydney, Ultimo, NSW 20227, Australia; 7 Department of Pharmacy, Faculty of Medicine and Pharmacy, University of Oradea, 410028 Oradea, Romania; 8 Doctoral School of Biological and Biomedical Sciences, University of Oradea, 410087 Oradea, Romania; 9 Department of Medical Disciplines, Faculty of Medicine and Pharmacy, University of Oradea, 410073 Oradea, Romania; briscristina@yahoo.com

**Keywords:** geophysics, medical imaging, artificial intelligence, machine learning, health sciences

## Abstract

To develop diagnostic imaging approaches, this paper emphasizes the transformational potential of merging geophysics with health sciences. Diagnostic imaging technology improvements have transformed the health sciences by enabling earlier and more precise disease identification, individualized therapy, and improved patient care. This review article examines the connection between geophysics and diagnostic imaging in the field of health sciences. Geophysics, which is typically used to explore Earth’s subsurface, has provided new uses of its methodology in the medical field, providing innovative solutions to pressing medical problems. The article examines the different geophysical techniques like electrical imaging, seismic imaging, and geophysics and their corresponding imaging techniques used in health sciences like tomography, magnetic resonance imaging, ultrasound imaging, etc. The examination includes the description, similarities, differences, and challenges associated with these techniques and how modified geophysical techniques can be used in imaging methods in health sciences. Examining the progression of each method from geophysics to medical imaging and its contributions to illness diagnosis, treatment planning, and monitoring are highlighted. Also, the utilization of geophysical data analysis techniques like signal processing and inversion techniques in image processing in health sciences has been briefly explained, along with different mathematical and computational tools in geophysics and how they can be implemented for image processing in health sciences. The key findings include the development of machine learning and artificial intelligence in geophysics-driven medical imaging, demonstrating the revolutionary effects of data-driven methods on precision, speed, and predictive modeling.

## 1. Introduction

Exploring the physical characteristics and mechanisms of the interior of the planet and its interactions with various natural phenomena is the focus of Earth science’s field of geophysics. It involves using physics concepts to examine Earth’s composition, structure, and dynamics. Seismic waves, electromagnetic fields, gravity, and magnetic fields are just a few of the techniques that geophysicists employ to learn more about Earth’s interior and its different layers [1]. Fundamentally, geophysics acts as a link between the Earth sciences and the physical sciences. Geophysics provides essential insights below and beyond our line of sight. Geophysicists have revealed the stratified nature of Earth’s interior, from the molten core to the solid crust, by painstakingly analyzing seismic waves from earthquakes [2]. Maps of subsurface features have been produced using gravity and magnetic surveys, assisting resource exploration and providing a better knowledge of tectonic plate movements. Additionally, geophysical techniques allow us to explore the exteriors and innards of other celestial bodies in addition to our planet [3].

Geophysics has a significant role in many parts of contemporary life. Natural resource exploration is one of its major contributions [4]. Geophysical technologies support sustainable resource management and economic growth by locating mineral deposits, groundwater reservoirs, and hydrocarbon reserves [5,6]. Additionally, geophysics is essential for hazard reduction. Scientists can better forecast earthquakes, tsunamis, and volcanic eruptions by keeping an eye on seismic activity, ground deformations, and volcanic processes [7]. This will protect communities and infrastructure [6].

Our understanding of Earth’s dynamic history is also based on geophysics. Geophysicists have contributed to the understanding of the history of Earth’s magnetic field reversals and the migration of continents over millions of years by examining the magnetic characteristics of rocks [8]. Geophysical methods also aid environmental research by allowing for the monitoring of land subsidence brought on by human activity, the evaluation of soil and water contamination, and the tracking of groundwater movement [9,10]. Geophysics has expanded outside its conventional fields in recent years, finding use in unexpected fields. Notably, its concepts have been applied in the field of health sciences [11,12], where methods like MRI use principles of strong magnetic fields, which had their origins in geophysics, to provide remarkably detailed images of internal human bodily structures [13,14].

In essence, geophysics is an invaluable tool for understanding the inner workings of Earth and solving the mysteries that exist underneath its surface. Natural resource management, emergency preparedness, environmental protection, and scientific research are all impacted by it [15]. The traditional uses of geophysics have been summarized in Table 1.

In the twenty-first century, numerous multidisciplinary studies have grown significantly, with one fascinating and revolutionary partnership arising between the historically separate domains of geophysics and health sciences. This confluence shows an increasing understanding that the ideas and methods produced in one area can be imaginatively used to address problems in another, frequently leading to ground-breaking answers that go beyond the bounds of traditional disciplines. Due to their mutual desire to discover unknown domains, geophysics and health sciences have recently begun to intersect significantly. Utilizing imaging technologies has allowed geophysics, historically linked with exploring Earth’s interior, and health sciences, which study human physiology, to collaborate more effectively. These tools, honed in the field of geophysics, have been redesigned to reveal the inside architecture and functioning of the human body in unparalleled detail [12].

The use of methods like magnetic resonance imaging (MRI) is an illustration of the growing collaboration between geophysics and the health sciences. Strong magnetic fields are used in MRI to provide precise images of soft tissues and organs. MRI was first designed to map geological features beneath the surface of Earth. Through non-invasive visualization of interior anatomy, early illness identification, and surgical intervention guidance, this interdisciplinary synergy has revolutionized diagnostic medicine [24].

**Figure 1:**
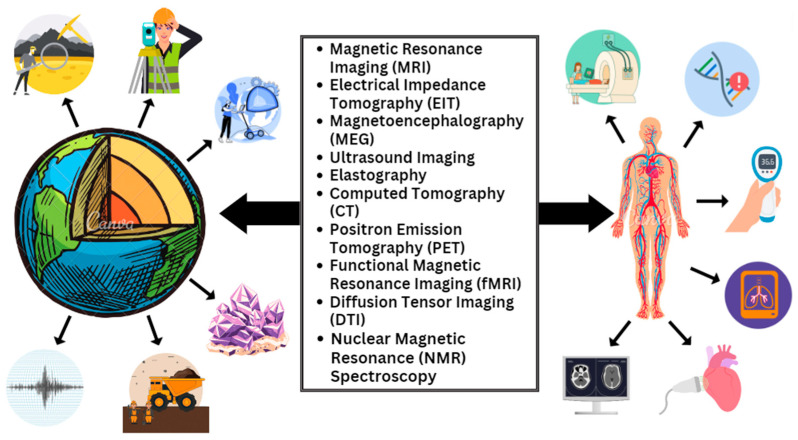
Various techniques that are used currently for diagnostic and imaging purposes in health sciences work on the same principles as those applicable to geophysics.

Additionally, the similarities in characteristics between biological tissues and geological formations have inspired creative uses [25]. For instance, the geophysical technique known as electrical impedance tomography (EIT), which is used to map differences in subsurface electrical conductivity, has been reimagined to capture changes in electrical conductivity inside the human body. This method shows the power of multidisciplinary borrowing by showing promise in monitoring lung function and finding anomalies. Moreover, the fusion of geophysics and health sciences solves typical computational issues. Both disciplines struggle with huge datasets and complex data processing requirements. For the correct and effective reconstruction of medical pictures, computational geophysical techniques like tomography and inversion techniques are being used [26]. Simultaneously, advances in the interpretation of geophysical data are being driven by the complex algorithms created in the health sciences, such as ML for medical picture analysis [27]. Figure 1 shows that diagnostic and imaging techniques in health sciences share fundamental principles with geophysics methods [12,27,28,29].

The continued partnership between geophysics and the health sciences emphasizes how transformational interdisciplinary research may be. It involves encouraging a creative interchange of ideas, processes, and insights rather than merely translating techniques from one discipline to another. This convergence is expanding the parameters of scientific inquiry and fostering a cross-disciplinary problem-solving culture. We can anticipate new diagnostic tools, improved treatment approaches, and a deeper knowledge of both Earth’s secrets and human well-being as geophysics and health sciences continue to collaborate. The review article focuses on:

- The principles and operating processes behind various geophysical techniques used in healthcare.
- Geophysical techniques that can be adapted and used in imaging techniques.
- The incorporation of artificial intelligence (AI) and machine learning (ML) for technological advancement in geophysics-driven medical imaging.

**Figure 2:**
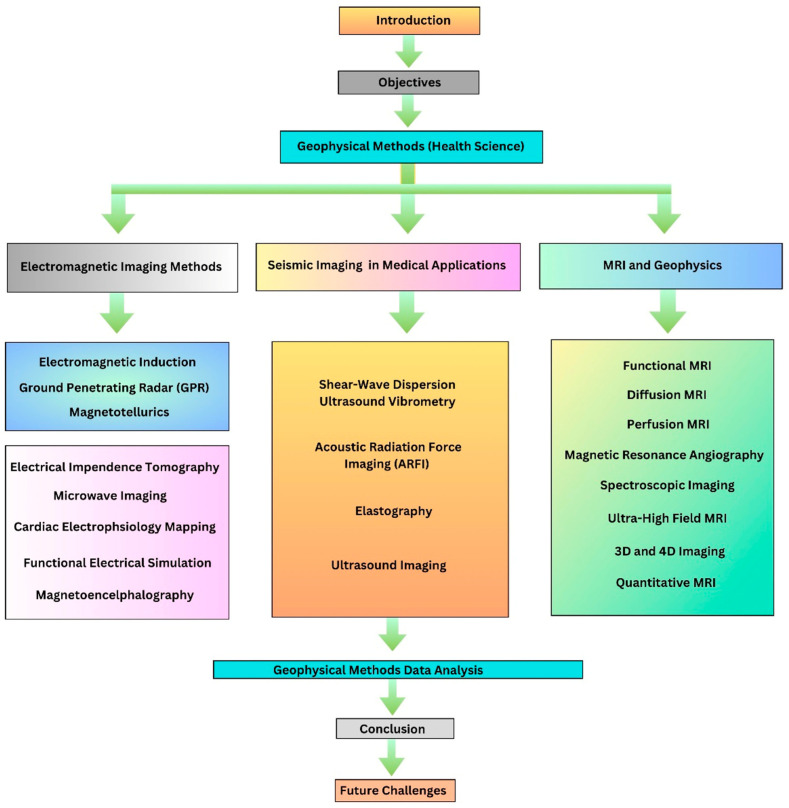
Various geophysical techniques and their applications in health sciences for diagnostic imaging such as FES, ARFI, magnetic resonance angiography, etc.

To fulfill the above outlined objectives, the flow of the article is shown in Figure 2.

## 2. Geophysical Methods in Health Sciences

The field of health sciences has been explored through geophysical techniques, which have historically been used to reveal the secrets of Earth’s subsurface. These methods have been cleverly repurposed to see inside the complex landscapes of the human body. They were originally developed to decipher geological features and uncover hidden treasures. A transformational synergy has developed by fusing the seemingly unrelated fields of geophysics and health, providing non-invasive methods for imaging medical problems, tracking physiological processes, and solving medical mysteries [12]. Several techniques currently used in health sciences have their principles derived from geophysics and have been listed in Table 2.

The opportunity for interdisciplinary study between geophysical methods and health sciences is enormous and growing all the time. Scientists are discovering novel approaches to improve medical diagnoses, solve physiological puzzles, and advance our understanding of human health and well-being as they investigate how adaptable these methods are [12].

### 2.1. Electromagnetic Imaging Method

Electromagnetic methods in geophysics use electromagnetic waves to collect data on the characteristics of Earth’s subsurface. These techniques use electromagnetic field interactions with materials to infer information on the underlying composition, conductivity, and structures of Earth [47]. The foundation of electromagnetic techniques is the idea that various materials respond differently to electromagnetic waves with different frequencies. Geophysicists can generate representations or models of the subsurface by monitoring these responses and assisting in geological exploration, resource discovery, and environmental studies [48].

Faraday’s law of electromagnetic induction and Ampère’s law, which states that electric currents produce magnetic fields, serve as the foundations of electromagnetic survey methodologies [49]. Faraday’s law, which can be expressed in its most basic form as “a changing magnetic field will induce an EMF,” asserts that the electromotive force (EMF) in a closed circuit is proportional to the rate of change of magnetic flux through the circuit [50].

The magnetic flux ( ∅ *B*) crossing a closed loop is mathematically expressed as [51]:

$$\emptyset B=\int_{area\;}\vec{B}.\;Ň\;d\vec{a}$$

where Ň is the outward pointing normal vector for the loop and $\vec{B}$ is the magnetic flux density, which is proportional to the magnetic field in free space.

Faraday’s law relates the magnetic flux through the surface bordered by the loop to the induced EMF in the loop [52]:

$$V=-\frac{d\emptyset B}{dt}$$

The current I’ flowing in the wire is related to the EMF through Ohm’s law:

V = IR,

where R is the electrical resistance of the circuit.

**Figure 3:**
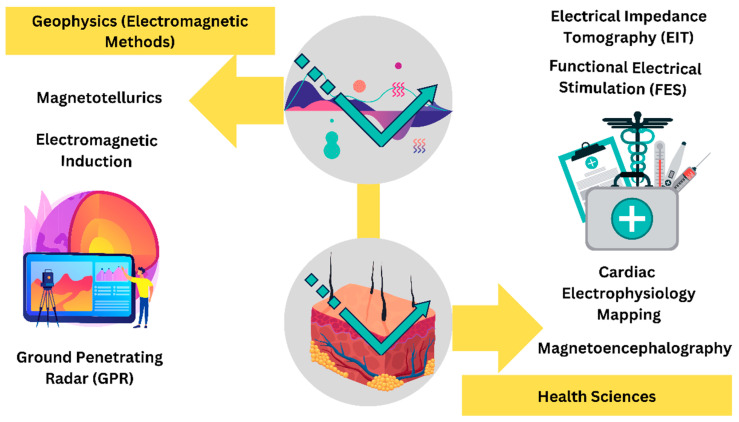
Use of different electromagnetic methods such as electromagnetic induction, GPR, and MT and their utilization for electromagnetic imaging EIT, FES, cardiac electrophysiology, and MEG in health sciences.

Some of the common electromagnetic methods in geophysics have been listed in Table 3, and the integration of electromagnetic methods in health sciences has been displayed in Figure 3.

It has been a revolutionary convergence of disciplines when electromagnetic techniques from geophysics have been used in the health sciences. These techniques, which were first created to investigate Earth’s subsurface, have been imaginatively repurposed to reveal complex bodily landscapes [12]. These methods have found use in physiological research, neuroscience, and medical diagnostics by taking advantage of interactions between electromagnetic waves and biological tissues, and the common methods are listed in Table 4.

### 2.2. Seismic Imaging in Medical Applications

Seismic waves, which are produced by controlled sources like explosions or vibrators and recorded by sensors called geophones or seismometers, are used in seismic imaging in the context of geophysics [66]. These waves interact with diverse geological features and formations as they move through Earth’s subsurface. The created images of the subsurface, which reveal important details about its composition, structure, and the presence of resources like oil, gas, and minerals, are created from the recorded seismic data. The principles of wave reflection, refraction, and transmission underlie seismic imaging [67].

Seismic waves experience variations in speed and direction when they encounter subsurface boundaries that have different qualities (such as density, elasticity, and rock type). Some of the waves are reflected back to the surface as a result of these modifications, and the sensors capture these reflections. Geophysicists can determine the features of subsurface layers and formations by examining the arrival times and amplitudes of these reflected waves [68]. Seismic profiles or sections are produced by combining data from various geophones or seismometers in seismic imaging. With the help of these profiles, you can see strata, faults, folds, and other geological characteristics in two dimensions [69]. Data are collected from different angles using more sophisticated techniques, such as 3D seismic imaging, to provide three-dimensional images that offer even more insight into subsurface structures.

In the seismic method, the seismic velocity model can be estimated to image some part of Earth’s subsurface given the condition that the modeled travel time matches with the observed seismic travel times, and the expression is given by [70]:

$$t\left(ray\right)=\int_{ray}s\left(x,z\right)dl$$

Such that,

*v*(*x*,*z*) = 2D velocity field
$s\left(x,z\right)=\frac{1}{v}(x,z)$ = reciprocal velocity
*dl* = differential distance along the ray

For the estimation of velocity, the difference between the observed and modeled travel times is minimized and the equation is given by:

∆t=D∆s

where ∆t = travel time difference vector, *D* = ray distance value matrix, and *S* = slowness vector.

Seismic imaging, which has historically been used to study Earth’s subsurface, has developed a distinctive and ground-breaking use in the field of medical sciences. Techniques for seismic imaging have been inventively repurposed from geophysics to depict the complex dynamics and features of the human body. Seismic imaging, used in the medical sciences, provides a non-invasive and in-depth image of inside tissues and organs by utilizing the principles of wave propagation and reflection [71]. This interdisciplinary convergence has paved the path for new diagnostic capabilities, allowing healthcare providers to access previously unavailable insights. A deeper comprehension of human anatomy, better illness detection, and more precise treatment planning are all made possible by seismic imaging, which continues to close the gap between geophysics and medicine. This synergy between two seemingly unrelated scientific fields is truly remarkable [12].

**Figure 4:**
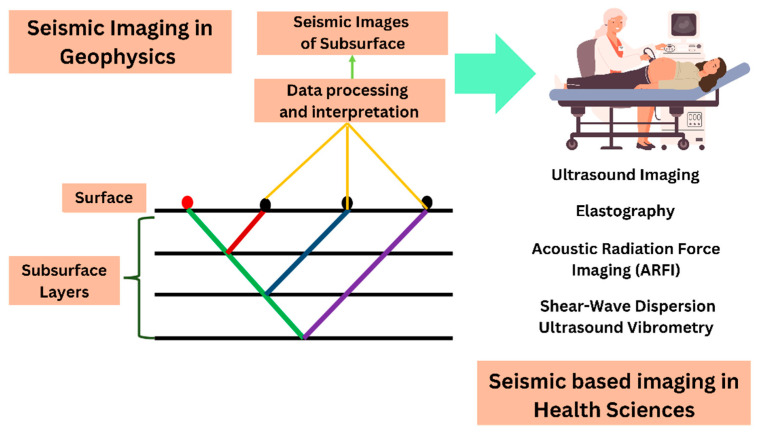
Use of seismic reflection techniques in diagnostic imaging such as ultrasound imaging, elastography, ARFI, and shear-wave dispersion ultrasound vibrometer.

Figure 4 illustrates the application of seismic reflection techniques in diagnostic imaging, including ultrasound imaging, elastography, ARFI, and shear-wave dispersion ultrasound vibrometer [12,67,72]. Moreover, the different techniques that have been derived from seismic images in geophysics for health sciences have been listed in Table 5.

### 2.3. MRI and Geophysics

The basic concepts of magnetic fields and signal detection are fundamental to both geophysics and MRI. They both use the interaction between magnetic fields and matter to extract useful information, despite having differing functions. By examining how various materials react to magnetic signals, geophysicists may investigate Earth’s subsurface and learn about its structures and characteristics. In contrast, MRI uses radiofrequency pulses and strong magnetic fields to provide precise images of the human body’s internal structures that highlight variations in tissue composition. Although their scales and goals are different, the fact that they both use magnetic interactions to probe matter highlights how closely related they are [81,82].

NMR is a concept that MRI technology uses in the field of medical imaging [83]. It entails exposing the human body to a strong magnetic field that is frequently tens of thousands of times stronger than the magnetic field of Earth. The hydrogen atoms in the body’s tissues have their nuclear spins aligned by this powerful magnetic field. The hydrogen nuclei absorb energy when a radiofrequency pulse is applied, and as they realign themselves, they begin to transmit signals. These “resonances” or signals are picked up by sensors and utilized to build extremely precise pictures of the insides of organs, blood arteries, and even the complicated networks of the brain [84,85].

The mutual reliance on powerful magnetic fields is the most overt sign of the relationship between MRI and geophysical principles. Both fields are aware of the special characteristics of magnetic materials and how they react to outside magnetic fields. Geophysics uses measurements of fluctuations in Earth’s magnetic field to deduce subsurface geological formations. Like this, MRI uses the interaction of powerful magnetic fields with tissue hydrogen nuclei to produce the signals that make up medical imaging. The applications and results, however, differ greatly [86]. Understanding Earth’s past, finding important resources, and foreseeing geological dangers are the objectives of geophysics [87]. In contrast, MRI focuses on non-invasively visualizing the human body’s internal structure to detect disorders, track the effectiveness of treatment, and direct surgical procedures. In addition to demonstrating the adaptability of magnetic interactions, the relationship between MRI and geophysical principles also emphasizes the interdisciplinary aspect of scientific advancement. It emphasizes how ideas from one field can be imaginatively modified to address problems in another. Strong magnetic fields are used in MRI and geophysics, which is an example of how research may go beyond preconceived bounds and benefit both human health and our comprehension of the natural world. The field of medical diagnostics has undergone a profound transformation because of improvements in MRI techniques, which have made it possible to more precisely and thoroughly visualize interior organs and bodily processes [13]. These developments have had a substantial impact on how different medical illnesses are diagnosed, enabling earlier detection, more accurate characterization, and better patient outcomes. Several advanced techniques have been listed in Table 6.

By providing innovative methods for detecting a variety of ailments, from neurological problems to cardiovascular diseases, oncology, and beyond, MRI techniques promise to make even bigger contributions to medical science as they continue to advance. The future of healthcare is extremely promising when personalized medication, new imaging techniques, and AI-driven methods are combined [101].

## 3. Geophysical Data Analysis in the Health Sciences

Geophysics has many applications in various fields, including in the diagnostic sphere of the medical sector [96,97], and some of the focal points are energy efficiency and sustainable development [98,99].

One key area where geophysical data analysis is making an impact in medical contexts is the analysis of biomedical signals. Techniques such as signal processing, time-series analysis, and spectral analysis, which are commonly used in geophysics to analyze seismic, electromagnetic, and other data, have been adapted to analyze medical signals like ECGs, EEGs, and even molecular data from gene expression studies [102]. These methods help identify patterns, anomalies, and trends within complex medical signals, aiding in the diagnosis of cardiac arrhythmias, neurological disorders, and genetic diseases [103].

The study of biomedical signals is one important area where geophysical data analysis has an impact in medical contexts. The analysis of medical signals like electrocardiograms ECGs, EEGs, and even molecular data from gene expression studies is now possible using methods like signal processing, time-series analysis, and spectral analysis, which are frequently used in geophysics to analyze seismic, electromagnetic, and other data. These techniques assist in the detection of cardiac arrhythmias, neurological illnesses, and genetic diseases by locating patterns, abnormalities, and trends within complex medical data [104,105].

In addition, medical imaging uses geophysical inversion techniques, which are utilized to retrieve subsurface properties from observed data. These techniques are used in medical imaging to recreate images of internal structures from a variety of modalities, including CT, MRI, and ultrasound [106]. The inversion techniques contribute to more precise and detailed medical imaging by enhancing spatial resolution, reducing artifacts, and improving image quality [107].

The examination of biological processes at the molecular and cellular level using geophysical principles in the science of biophysics is another interesting application. Examples include the use of methods like NMR spectroscopy, which was initially created to study the behavior of atomic nuclei in Earth materials, to examine the structure and dynamics of biological molecules like proteins and nucleic acids. This clarifies molecular connections, assisting in the development of new drugs and the comprehension of disease mechanisms [107,108,109].

The similarities in the mathematical and statistical techniques needed to glean useful information from large, complex datasets underlie the relationship between geophysical data analysis and medical situations. The algorithms, models, and tools created in geophysics are being repurposed to analyze medical data as data-driven approaches become more common in medicine, opening up new perspectives and opportunities [110,111]. The accuracy of data analysis depends on reliable procedures, cutting-edge algorithms, and experts in both geophysics and medical imaging. Erroneous conclusions can result from errors or misinterpretations, which may result in missed opportunities or poor medical decisions. The significance of proper data analysis increases as technology develops because the complexity of data necessitates advanced approaches to extract insightful information [112,113].

The analysis and interpretation of complicated medical data have benefited from the use of computational tools that were initially created for processing geophysical data. These methods take advantage of the similarities between data analysis, signal processing, and image reconstruction to provide insights that lead to more precise diagnoses, individualized therapies, and better patient care [114]. In the field of health sciences, the following geophysical analysis procedures are now in use:

- Signal Processing: ECG, EEGs, EEGs, and even functional MRI data are treated as medical signals using signal processing techniques utilized in geophysics, including filtering, noise reduction, and Fourier analysis. These methods aid in pattern recognition, abnormality quantification, and information extraction from noisy data [115,116].
- Image Reconstruction: Medical imaging modalities like CT and MRI are developed from geophysical data inversion methods, which are used to rebuild subsurface structures. These techniques produce more realistic reconstructions of anatomical features by improving image quality, lowering artifacts, and boosting spatial resolution [117].
- Data Fusion: Medical data fusion uses methods for integrating various geophysical information to provide a full perspective of subsurface features. Combining data from various imaging modalities (such as MRI and PET) in medical imaging allows for a more thorough understanding of the interior organs and their activities [118,119].
- ML and AI: Medical imaging uses tomographic techniques to recreate precise images of organs and tissues. Tomographic techniques are frequently utilized in geophysics to build 3D images of subsurface objects. Examples include X-ray CT and PET scans, where cross-sectional images are produced using information from various angles [120,121,122,123].
- Tomographic Imaging: Medical imaging uses tomographic techniques to recreate precise images of organs and tissues. Tomographic techniques are frequently utilized in geophysics to build 3D images of subsurface objects. Examples include X-ray CT and PET scans, where cross-sectional images are produced using information from various angles [124].
- Inverse Problem Solving: Techniques for inverse problem-solving are used to recover data from imperfect or indirect measurements in both geophysics and medicine. This aids in the discovery of subsurface features in geophysics and the reconstruction of images from sparse data in medicine, such as in magnetic resonance spectroscopy (MRS) for the investigation of metabolic processes [122].
- Quantitative Analysis: To measure qualities such as tissue density and blood flow in medical imaging, computational approaches that quantify variables like density, composition, and electrical conductivity in geophysics are modified [125,126].
- Image Registration: To register medical pictures from various periods or modalities, geophysical techniques that align and match multiple datasets are used. This aids in monitoring illness development, evaluating therapy effectiveness, and directing interventions [127,128].

The application of geophysical computational methods in the field of medicine emphasizes the interdisciplinary aspect of contemporary science. These methods not only improve the accuracy of medical data analysis but also offer a fresh viewpoint on medical problems [129]. Medical researchers can make use of the developments achieved in geophysics by modifying and extending computational tools to push the boundaries of medical diagnostics, treatment approaches, and our comprehension of the vast complexity of the human body. This interdisciplinary cooperation enhances diagnostic precision, showcasing the broader implications of technology transfer for advancements in medical research and patient care [128,129].

### Artificial Intelligence (AI) and Machine Learning (ML) Applications

AI and ML have significantly transformed medical imaging by advancing data analysis across various modalities (Table 7). In diagnostic radiology, AI algorithms enhance the interpretation of X-rays, CT scans, and MRIs, accelerating the identification of abnormalities and streamlining diagnostics. Computer-aided diagnosis (CAD) systems, powered by AI, play a crucial role in mammography, aiding early detection of breast cancer by analyzing mammograms and assisting radiologists in making more precise diagnoses. The application of AI extends to cardiac imaging, where ML is utilized to detect cardiovascular diseases and abnormalities in heart function through the analysis of echocardiograms and cardiac MRIs. In pathology, ML contributes to image analysis, supporting pathologists in diagnosing diseases, particularly cancer, by examining tissue samples and identifying specific markers or abnormalities [130].

Neuroimaging benefits from AI, which aids in mapping brain activity, identifying neurological disorders, and studying brain connectivity using functional MRI (fMRI) and diffusion tensor imaging (DTI). Ophthalmology leverages AI for the analysis of retinal images, enabling the detection and monitoring of conditions like diabetic retinopathy [131]. Positron emission tomography (PET) imaging sees AI analysis for cancer diagnosis and staging, enhancing the identification and characterization of tumor activity. Ultrasound imaging benefits from ML applications, improving accuracy in fetal imaging, organ recognition, and abnormality detection across various medical specialties. Quantitative image analysis, facilitated by AI, provides objective measurements of features such as tumor size and density, contributing to cancer research and treatment monitoring [132,133].

The integration of medical imaging data with genomic information is achieved through AI, enabling a comprehensive understanding of diseases and supporting personalized treatment strategies. These diverse applications underscore the transformative potential of AI and ML in enhancing diagnostic precision, efficiency, and patient outcomes in the dynamic field of medical imaging. Staying informed about ongoing research is crucial to keeping pace with advancements in this rapidly evolving domain. ML and AI are related but distinct fields, and their separation is meaningful in the context of health sciences. ML, a subset of AI, focuses on creating algorithms that enable computers to perform tasks without explicit programming, learning and improving from data or experience [134].

In health sciences, ML is extensively applied for predictive modeling, classification, and pattern recognition, contributing to tasks such as diagnosing diseases, predicting patient outcomes, and identifying risk factors. On the other hand, AI is a broader concept encompassing various technologies beyond ML, including natural language processing, robotics, and expert systems. In healthcare, AI extends beyond ML to include reasoning, problem-solving, and decision-making capabilities, providing a comprehensive approach to improving diagnostics, treatment plans, and healthcare management. The separation of ML and AI allows for a nuanced understanding, acknowledging ML’s specific role in learning from data and AI’s broader spectrum of technologies that contribute to intelligent decision making in health sciences [135].

**Table 7:** Various algorithms and their applications in data analysis for health sciences [136,137,138,139,140,141].

Algorithm	Description
Linear Regression	Application involves predicting patient outcomes based on clinical parameters, such as age, blood pressure, and cholesterol levels. In clinical trials, linear regression aids in analyzing the relationship between treatment dosage and response. The method is also employed for assessing risk factors associated with specific health conditions and modeling disease progression over time. Public health studies benefit from linear regression to understand how environmental factors impact population health.
Logistic Regression	Logistic regression can be utilized to assess the probability of a patient developing a particular disease based on various risk factors such as age, genetic predisposition, and lifestyle choices. This method is instrumental in risk assessment and enables healthcare professionals to identify significant predictors for targeted interventions. Logistic regression is also employed in clinical research, analyzing factors influencing treatment success or failure.
Decision Tree and Random Forest	Decision trees, being intuitive and easy to interpret, are often applied to predict outcomes such as disease presence or treatment response. For instance, a decision tree might be constructed to assess the likelihood of a patient developing a specific condition based on various medical parameters. Random forests, which are ensembles of decision trees, offer enhanced predictive performance and robustness. In health sciences, random forests find applications in scenarios where complex relationships among multiple variables influence health outcomes. For example, in genomics, random forests can be used to identify genetic markers associated with certain diseases by analyzing large datasets.
Support Vector Machines (SVMs)	SVMs find applications in identifying patterns within genetic data, aiding in the classification of patients into distinct subgroups based on their genetic profiles. Additionally, SVMs contribute to personalized medicine by assisting in the prediction of treatment responses. SVMs can be utilized to discern between different medical conditions based on patient data, facilitating accurate disease diagnosis. Their ability to handle high-dimensional data and nonlinear relationships makes SVMs suitable for complex medical datasets.
K-Nearest Neighbor (KNN)	KNN is particularly useful in personalized medicine, where it aids in recommending treatments based on the experiences of similar patients. It considers the proximity of an individual’s health profile to others, offering tailored insights for healthcare interventions. Additionally, KNN finds applications in epidemiology, helping identify clusters of diseases or health conditions within a population.
Naive Bayes	Naive Bayes is employed for tasks such as disease diagnosis and risk assessment. For instance, it can be applied to evaluate the probability of a patient having a specific medical condition given certain observable symptoms, aiding in efficient and accurate diagnosis. Naive Bayes is commonly used in analyzing electronic health records, patient demographics, and diagnostic test results.
K-Means Clustering	This algorithm is commonly used to identify natural groupings or clusters within datasets, enabling the segmentation of patients based on similar characteristics. In healthcare, K-means clustering is applied to stratify patient populations, allowing for personalized treatment approaches and targeted interventions. One notable application involves patient segmentation for risk assessment. K-means clustering can group patients with similar health profiles, aiding healthcare professionals in identifying high-risk populations that may require specific monitoring or preventive measures.
Principal Component Analysis (PCA)	AI-driven methods help automate image interpretation, improve image quality, and shorten scan times. AI tools help radiologists spot problems and boost their level of diagnostic assurance.
Convolutional Neural Networks (CNNs)	CNNs are adept at extracting intricate patterns and features from visual data, making them invaluable for image classification and recognition. In healthcare, CNNs are extensively applied to analyze medical images such as X-rays, MRIs, and CT scans, aiding in the detection and classification of abnormalities. One significant application involves the automated diagnosis of diseases based on medical images. CNNs excel at discerning subtle patterns indicative of various conditions, contributing to faster and more accurate diagnoses. For instance, in radiology, CNNs can assist in identifying tumors, fractures, or anomalies in scans, enhancing the efficiency. CNNs are employed in pathology image analysis, where they can assist in the classification of tissue samples by identifying cellular structures and anomalies.
Recurrent Neural Networks (RNNs)	RNNs can learn temporal dependencies and patterns in a patient’s medical history, facilitating the prediction of disease progression or the likelihood of future health events. RNNs contribute to personalized medicine by tailoring predictions based on individual patient data. They are adept at capturing the dynamic nature of health-related data, allowing for more accurate predictions and timely interventions.

## 4. Conclusions

The review has shown how extraordinary a role geophysics can play in advancing diagnostic imaging methods within the field of health sciences. Ingenious solutions to enduring medical problems have been made possible by the cooperation between two otherwise disparate professions. The cross-disciplinary partnership has produced game-changing outcomes, from the adaption of electromagnetic tools for imaging biological tissues to the introduction of geophysical computing techniques into medical analysis.

Applications like EIT, MEG, FES, cardiac electrophysiology mapping, and microwave imaging, taken as a whole, demonstrate the adaptability of geophysical techniques in transforming medical diagnostics, patient care, and our understanding of complex physiological processes. By combining the domains of geophysics and health sciences, it has been shown that the fundamental ideas of wave propagation, signal analysis, and data interpretation cut across disciplinary barriers and can provide creative answers in both areas.

Additionally, the rise of AI and ML has given this interdisciplinary partnership new life and holds the potential to speed up improvements in data analysis, picture reconstruction, and predictive modeling. As we look to the future, it is expected that the fusion of geophysics and health sciences will continue to open up new vistas, improving diagnostic precision, personalizing treatment plans, and advancing the field of precision medicine.

It is crucial to navigate the difficulties of adjusting geophysical techniques to biological contexts, ensuring patient safety, addressing ethical issues, and encouraging partnerships between these various fields as researchers, clinicians, and scientists continue to forge ahead in this dynamic environment. The review culminates by highlighting the transformative potential of interdisciplinary inquiry and the significant influence geophysics has had and will continue to have on the development of diagnostic imaging methods in the health sciences.

## 5. Future Challenges and Scopes

**Figure 5:**
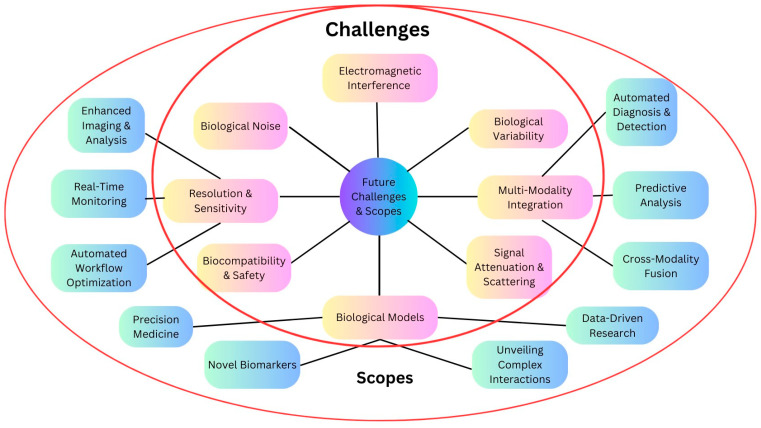
Use of different electromagnetic methods such as electromagnetic induction, GPR, and MT and their utilization for electromagnetic imaging (EIT-EIT, FES-FES, cardiac electrophysiology, and MEG) in health sciences.

The change from researching geological materials to living tissues poses a special set of problems for the use of geophysical approaches in the health sciences. While the fundamentals of wave propagation and data analysis are still valid, the complexity of living systems poses various challenges that must be overcome to obtain accurate and useful results. Figure 5 and Table 8 and Table 9 show the future challenges and scopes for the implementation of geophysics in the medical field [12,29,142,143,144].

Despite these difficulties, using geophysical methods in the field of health sciences has significant potential benefits. By overcoming these obstacles, we can revolutionize medical diagnostics, treatment monitoring, and our understanding of physiological processes. This will require novel technology, computational advances, and interdisciplinary collaboration. Transformational advances in patient care and scientific discovery will surely result from the connection between geophysics and health sciences as these challenges are met.

Geophysics-driven medical imaging has significant potential for the integration of ML and AI in the future, opening the door for previously unimaginable improvements in patient care, diagnosis, and therapy.

## Figures and Tables

**Table 1:** Different applications of geophysics for studying different Earth processes.

Application	Description	Refs.
Seismology	The study of seismic waves produced by earthquakes or other sources is known as seismology. These waves reveal details about the composition of Earth’s inner layers and the characteristics of the materials that constitute them.	[16]
Gravity and magnetic surveys	Geophysicists can locate subsurface features like faults, mineral deposits, and even underground water reservoirs by detecting fluctuations in Earth’s gravitational and magnetic fields.	[17]
Electromagnetic methods	To determine the electrical conductivity and other characteristics of the subsurface, electromagnetic techniques such as magneto-telluric examine fluctuations in Earth’s electromagnetic field.	[18]
Geodesy	The goal of geodesy is to precisely measure the gravitational field, shape, and rotation of Earth. For mapping, navigation, and a general understanding of Earth dynamics, this information is essential.	[19]
Geothermal studies	To investigate the distribution of heat on Earth and the possibility of geothermal energy production, geophysicists employ a variety of techniques.	[20]
Natural resources exploration	By identifying abnormalities in the subsurface, geophysics is essential in locating important resources like oil, gas, minerals, and groundwater.	[8]
Volcanology	Researchers can keep track of and better understand the mechanisms underlying volcanic eruptions, reducing the risks associated with them by examining seismic activity, ground deformation, and other geophysical indicators.	[21]
Tectonics and plate movements	Our understanding of tectonic plate movement, the forces that propel it, and the geological events that emerge from it, such as earthquakes and mountain development, is aided by geophysics.	[22]
Environmental studies	To research how human activity affects the planet, geophysical techniques are used, including monitoring changes in the surface and subsurface of the planet as a result of things like groundwater extraction, land subsidence, and more.	[23]

**Table 2:** Different medical techniques that have their origins in geophysics.

Technique	Description	Refs.
Magnetic Resonance Imaging (MRI)	MRI has completely changed medical imaging. Its foundations lay in the concepts of strong magnetic fields employed in geophysics. It produces fine-grained pictures of soft tissues using strong magnets, enabling non-invasive visualization of anatomical features and pathologies inside the human body.	[30]
Electrical Impedance Tomography (EIT)	EIT has been modified to track alterations in electrical conductivity within the body. EIT is a technique traditionally used in geophysics to map subsurface electrical conductivity. It might be used for tissue property monitoring and lung imaging.	[31]
Magnetoencephalography (MEG)	MEG measures the magnetic fields produced by neural activity in the brain and is derived from techniques for detecting electromagnetic signals from Earth’s magnetic field. It is employed in neuroscience research and offers useful insights into how the brain functions.	[32]
Ultrasound Imaging	High-frequency sound waves are used in ultrasound procedures to produce images of soft tissues and organs. These techniques were inspired by seismic approaches. For real-time visualization, it is commonly utilized in obstetrics, cardiology, and other medical disciplines.	[33]
Elastography	Elastography techniques, which were adopted from seismic technologies, measure the movement of mechanical waves across the body to determine the stiffness of the tissue. These data are useful for identifying anomalies and evaluating tissue health.	[34]
Computerized Tomography (CT)	CT scans, which employ X-rays to create cross-sectional images of the body, are not directly geophysical methods. There are similarities between the reconstruction algorithms used in CT imaging and the tomographic methods used in geophysics.	[35,36]
Positron Emission Tomography (PET)	PET imaging picks up injected positron-emitting radionuclides. Although not explicitly a geophysics technique, the concepts of gamma-ray detection are comparable to those of the gamma-ray spectrometry used in geophysics.	[37,38]
Functional MRI (fMRI)	fMRI is an advancement above conventional MRI in that it maps brain activity by monitoring changes in blood flow and oxygenation. This method sheds light on the cognitive functions and functional connectivity of the brain.	[39]
Diffusion Tensor Imaging (DTI)	DTI, which is based on the diffusion of water molecules, is a technique used in neuroimaging to show the neuronal connections and pathways in the brain. Diffusion-based geophysical techniques serve as the basis for this strategy.	[40]
NMR Spectroscopy	NMR spectroscopy, which is derived from NMR methods used to study molecular structures and is utilized in medical settings to examine biological molecules, is an area of study in molecular biology and medicine.	[41]
Near-Infrared Spectroscopy (NIRS)	The NIRS technique evaluates the tissues’ near-infrared light absorption, which was first applied in geophysics to examine mineral composition. It is used in the health sciences to measure brain activity, monitor vital signs in neonatal care, and evaluate tissue oxygenation.	[42]
Photoacoustic Imaging	Photoacoustic imaging combines optical and ultrasonic approaches and is inspired by seismic methods. By sensing the acoustic waves that tissues emit when they absorb laser-generated light, it creates images. High-resolution imaging of tumors, blood arteries, and other structures is made possible by this method.	[43]
Fluorescence Imaging	Despite not being a direct geophysics tool, fluorescence imaging is analogous to how fluorescence is used in geology. Fluorescent molecules are employed to mark certain cells in medical settings, facilitating cellular imaging, cancer diagnosis, and therapeutic development.	[44]
Terahertz Imaging	Terahertz imaging uses terahertz radiation, which was first applied to astronomy and atmospheric research, to see inside biological tissues. This method may be used to evaluate tissue characteristics and detect skin malignancies.	[45]
Biomechanical Analysis	The ideas of analyzing material qualities and deformation in geophysics have influenced biomechanical analysis in the health sciences; however, this is not a specific geophysics technique. Biomechanical analysis includes determining how organs and tissues react to mechanical forces, assisting in the creation of orthotics and prostheses, and comprehending how people move.	[46]

**Table 3:** Summary of different electromagnetic methods used in geophysics.

Working	Principle	Applications	Mathematical Expressions	Refs.
**Electromagnetic Induction**				
A transmitter coil is used to produce a magnetic field that changes over time. Electrical currents are induced in conductive subsurface materials as a result. Receiver coils at the surface detect the secondary magnetic fields that are produced as a result of these induced currents.	Faraday’s law of electromagnetic induction	Groundwater exploration, mineral resource identification	E = − $\frac{\partial B}{\partial t}$ $\frac{1}{\sigma }$ . Where E is the induced electrical field, σ is electrical conductivity, and $\frac{\partial B}{\partial t}$ is the rate of change of the magnetic field.	[48,49]
**Ground Penetrating Radar (GPR)**				
Short electromagnetic pulses are released into the ground using GPR. The travel period of these pulses is measured after they reflect off subsurface contacts. Information about the depth and characteristics of subsurface features can be gleaned from the timing of the reflected waves.	EM wave reflection at material boundaries	Archaeology, environmental studies, detecting buried objects	d = $\frac{\vartheta t}{2}$ Where t is the time taken for the signal to travel to the subsurface and back, *ϑ* is the wave speed, and d is the depth.	[53,54]
**Magnetotellurics (MT)**				
Earth’s electromagnetic field naturally varies owing to interactions with the sun and other celestial bodies, as measured by MT. A variety of frequencies are used to record these fluctuations. Earth’s impedance response sheds light on changes in subsurface resistivity.	The penetration of electromagnetic waves is influenced by the conductivity of Earth.	Mapping subsurface structures, understanding geological formations	The ratio of the vertical electric field (*E_z_*) to the horizontal magnetic field (*H_y_*) is used to calculate the impedance (*Z*) *Z* = $\frac{Ez}{Hy}$	[20,55]

**Table 4:** Different medical techniques used in health sciences based on electromagnetic methods in geophysics.

Description	Working	Applications	Refs.
**Electrical Impedance Tomography (EIT)**			
When using EIT, which is a non-invasive imaging approach, tiny electrical currents are passed through biological tissues and the surface voltage changes that arise are then measured. The electrical conductivity distribution throughout the body can be precisely analyzed by EIT to yield specific information that, in turn, reveals important insights about the interior structures and physiological processes. Electrical techniques are employed in geophysics to examine Earth’s subsurface by observing changes in electrical conductivity. Similarly to this, EIT uses electrical conductivity’s fundamental concepts but applies them to medical imaging.	EIT involves applying electrode arrays to the surface of the body. The voltage distribution that results from the injection of a safe electric current into the body by these electrodes is monitored. EIT reconstructs a tomographic image of the internal conductivity distribution by utilizing various electrode configurations and examining the voltage variations. The anatomy, blood flow, ventilation, and even the ability to spot abnormalities like tumors or edema can all be learned from this image.	Lung imaging, brain and breast imaging	[31,56]
**Magnetotellurics (MEG)**			
Measuring the magnetic fields generated by neural activity in the brain is possible using MEG, a potent neuroimaging technology utilized in the health sciences. With remarkable temporal and spatial resolution, MEG offers useful insights into brain function, connection, and localization of brain activity. Interestingly, geophysics and the study of Earth’s magnetic field are where MEG gets its start. This method aids in mapping the electrical conductivity of Earth’s subsurface in geophysics. Similar to this, MEG uses electromagnetic induction’s basic principles to measure neural activity.	It operates by taking measurements of the weak magnetic fields generated by the electrical activity of brain cells. SQUIDs (superconducting quantum interference devices), the incredibly sensitive sensors used in MEG systems, are capable of detecting these minute magnetic fields. Small electrical currents are produced by activated brain neurons. Beyond the skull and scalp, these currents generate magnetic fields that relate to them. The head is surrounded by MEG sensors that are used to identify these magnetic fields. With exceptional temporal and spatial precision, MEG can map the brain’s activity by carefully examining the timing and distribution of these magnetic signals.	Detecting anomalies in the brain and assisting with pre-surgical planning for diseases like epilepsy	[57,58]
**Functional Electrical Stimulation (FES)**			[59,60]
FES (FES), a medical treatment used in the health sciences, uses regulated electrical currents to help paralyzed or weak muscles regain or improve their function. Electrical impulses are delivered to specific muscles or nerves during FES, prompting them to contract and produce movement. For people with diseases like spinal cord injuries, strokes, or neuromuscular abnormalities, this method is especially helpful. Although FES is not directly related to geophysics, its basic principles have some loose similarities to geophysical ideas. The fundamentals of electrical currents and their effects on materials are fundamental to both fields and electrical methods are utilized in geophysics to examine subsurface conductivity variations.	FES works by implanting electrodes near the target location or placing electrodes on the skin to provide regulated electrical pulses to muscles or nerves. These electrical impulses cause muscle contractions, enabling people with paralysis or weak muscles to carry out practical motions like standing or walking.	Stroke rehabilitation, neuromuscular disorders, orthopedic rehabilitation, pain management, neuroprosthetics	
**Cardiac Electrophysiology Mapping**			
Cardiac electrophysiology mapping is a sophisticated method used to visualize and examine the electrical activity of the heart. This approach assists in recognizing irregular heart rhythms (arrhythmias), locating their causes, and directing medical procedures like catheter ablation. Cardiac electrophysiology mapping entails mapping the electrical signals of the heart in great detail to diagnose and treat a variety of cardiac diseases. Cardiac electrophysiology mapping and geophysical methods can be broadly compared by understanding how electrical currents spread across a medium, although this is not directly related to geophysics.	To capture the electrical activity of the heart, electrode-equipped catheters are inserted into the organ. Electrical signals go through the heart’s chambers as it beats. These impulses are picked up by the electrodes, which produce fine-grained maps that depict the strength and order of the signals in various heart areas.	Arrhythmia diagnosis, catheter ablation guidance, ventricular tachycardia localization, atrial fibrillation management, Wolff–Parkinson–White syndrome, ventricular fibrillation risk assessment	[61,62]
**Microwave Imaging**			
A developing method in the health sciences called microwave imaging uses electromagnetic radiation in the microwave frequency range to produce images of biological tissues. To visualize internal structures and abnormalities, microwave imaging makes use of the interaction between tissues and microwaves to highlight changes in their dielectric characteristics. The similarities between electromagnetic wave propagation and imaging can be used to create connections between microwave imaging and geophysics. Electromagnetic techniques are used in geophysics to investigate material characteristics and underlying structures. The fundamental idea of employing electromagnetic waves to obtain data is present in both domains, despite the scales and circumstances being different.	This entails sending microwaves into the body and observing how they affect the tissues. Due to differences in composition, different tissues have unique dielectric characteristics. These tissues reflect, absorb, or disperse microwaves differently as they travel through the body. The changing microwave signals are captured by sensitive receivers and the data are processed by sophisticated algorithms to produce images that show the spread of dielectric characteristics.	Breast cancer detection, tumor monitoring, brain imaging, musculoskeletal imaging, monitoring of tissue changes, stroke detection	[63,64,65]

**Table 5:** Different medical techniques used in health sciences based on seismic images.

Description	Working	Applications	Refs.
**Ultrasound Imaging**			
Ultrasound imaging is a non-invasive, safe, and extensively used medical imaging technique that employs high-frequency sound waves (ultrasound) to produce real-time images of the body’s internal components. While geophysics studies Earth’s subsurface with seismic waves, ultrasonography visualizes internal structures with sound waves. To reveal concealed information, both areas rely on wave interactions, reflection, and imaging techniques.	The notion of sound wave reflection underpins ultrasound imaging. A transducer sends ultrasonic waves into the body that bounce off tissues and organs to create echoes. These echoes are received by the transducer and converted into electrical impulses. A computer then processes these impulses to generate visuals that are displayed on a monitor.	Obstetrics and gynecology, cardiology, abdominal imaging, musculoskeletal imaging, vascular imaging, breast imaging	[73,74]
**Elastography**			
Elastography is a type of medical imaging that assesses the stiffness or elasticity of tissues. It offers information about the mechanical properties of tissues, assisting in the identification of anomalies and assessing circumstances that affect tissue stiffness. In geophysics, seismic waves move through the subsurface of Earth and interact with various rock layers to disclose details about their characteristics. Like this, elastography interacts with tissues using mechanical waves, assesses their reaction, and reveals information about the characteristics of the tissues.	Elastography is the process of applying mechanical forces to tissues and monitoring how they respond. Elastography techniques include shear-wave elastography, strain elastography, and magnetic resonance elastography. Shear-wave elastography is commonly used to create shear waves within tissues using ultrasound or other mechanical techniques. The speed of these shear waves as they propagate through the tissue is measured. Shear waves are transmitted faster by stiffer tissues than by softer ones.	Cancer detection, liver fibrosis assessment, breast imaging, musculoskeletal disorders, cardiovascular health	[75,76,77]
**Acoustic Radiation Force Imaging (ARFI)**			
Focused ultrasound beams are utilized in this medical imaging approach to cause mechanical vibrations in the tissues. ARFI measures the ensuing tissue displacement to determine the stiffness of the tissue, which helps in the diagnosis of various medical diseases. Seismic waves interact with underground formations in geophysics and provide data on their characteristics. Similar to this, ARFI uses sonic waves to interact with tissues and then measures the response to infer information about the tissues. Both fields make use of the idea of wave interaction to learn more about the structure and properties of a medium.	Short bursts of extremely intense ultrasound waves are used in ARFI to penetrate the body. The acoustic radiation force exerted by these waves causes localized vibrations in the tissues. The displacement of tissue because of these vibrations is then detected by sensors. The amount of tissue displacement is directly correlated with the stiffness of the tissue: softer tissues exhibit greater displacement, whereas stiffer tissues exhibit less.	Liver disease assessment, breast imaging, musculoskeletal disorders	[78,79]
**Shear-Wave Dispersion Ultrasound Vibrometry**			
This is a cutting-edge medical imaging method that evaluates tissue stiffness by examining the shear-wave dispersion within tissues. It aids in the diagnosis of disorders like liver fibrosis by giving insights into the mechanical characteristics of tissues, particularly their viscoelastic behavior. Due to underlying characteristics, different kinds of seismic waves move at varying rates in geophysics. Seismic shear waves, for instance, show dispersion in geological formations. Like this, SDUV uses shear wave dispersion to give details about the mechanical characteristics of the tissue.	Focused ultrasound beams are emitted by SDUV to create shear waves inside tissues. Multiple frequencies are used to measure the pace at which these shear waves move through the tissues. Due to their viscoelastic characteristics, tissues respond to shear waves in different ways and at different frequencies. SDUV gives data on tissue stiffness and viscosity by examining the shear-wave speed dispersion across frequencies.	Liver fibrosis assessment, musculoskeletal disorders	[75,80]

**Table 6:** Advancements in MRI techniques.

Techniques	Description	Refs.
Functional MRI (fMRI)	fMRI uses blood flow fluctuations to pinpoint the brain’s active regions. It is essential to neuroscience and the comprehension of how the brain processes language, memory, and emotion. In clinical contexts, fMRI aids in the diagnosis of neurological illnesses, assists in mapping brain activity before surgery, and tracks therapeutic responses.	[88,89]
Diffusion MRI	This method evaluates how water molecules travel within tissues. It is extremely useful for neuroimaging, especially in the diagnosis of stroke and the investigation of white matter integrity. The use of diffusion MRI enables the early detection of brain injury and sheds light on neurodegenerative illnesses.	[90,91]
Perfusion MRI	Perfusion imaging analyzes blood flow to tissues, which helps in tumor evaluation and stroke detection. In ischemic strokes, it aids in separating viable from non-viable tissue and directs therapeutic choices.	[92,93]
Magnetic Resonance Angiography (MRA)	MRA reduces the requirement for contrast chemicals by non-invasively visualizing blood vessels. It is employed in determining aneurysms, evaluating vascular anomalies, and organizing interventions.	[94,95]
Spectroscopic Imaging	This method determines the chemical composition of tissues, allowing for metabolic evaluations. Spectroscopic imaging improves tumor characterization in oncology by distinguishing between malignant and healthy tissue.	[96,97]
3D and 4D Imaging	Modern imaging methods provide real-time, four-dimensional and three-dimensional views of organs and structures. This makes it easier to visualize dynamic movements, intricate anatomical linkages, and cardiac activity.	[85]
Ultra-High Field MRI	Greater spatial and temporal resolution is made possible by stronger magnetic fields (7T and higher). The visualization of small structures, such as blood arteries, joints, and the brain, is enhanced by ultra-high field MRI, which enhances diagnostic precision.	[83,84]
Quantitative MRI	By measuring particular tissue characteristics like T1 and T2 relaxation periods, this method makes it easier to distinguish between healthy and sick tissues. It helps determine the severity of diseases including multiple sclerosis and liver fibrosis.	[98]
AI Integration	AI-driven methods help automate image interpretation, improve image quality, and shorten scan times. AI tools help radiologists spot problems and boost their level of diagnostic assurance.	[99,100]

**Table 8:** Different challenges in the integration of geophysics to imaging methods in health sciences.

Challenges	Description
Biological variability	Because of variables like age, gender, heredity, and health problems, biological tissues are fundamentally varied. The interpretation of geophysical data may be impacted by this variability, necessitating the use of robust statistical techniques to take this into account and distinguish between normal and aberrant fluctuations
Resolution and sensitivity	Compared to geological materials, biological tissues are frequently more delicate and irregular in texture. It might be difficult to achieve great resolution and sensitivity while protecting tissue integrity. To spot tiny changes and anomalies without causing harm, techniques must be improved
Signal attenuation and scattering	Signals can attenuate and scatter differently in biological tissues compared with in geological ones. The accuracy of the data gathered may be impacted by diminished signal penetration and distortion as a result. Techniques must be improved to take tissue-specific signal behaviors into account
Electromagnetic interference	Electromagnetic interference can be introduced by biological tissues and affect data capture. To obtain accurate results, strategies to reduce or adjust for this interference are essential
Biocompatibility and safety	Patient safety is of the utmost concern. Strict safety guidelines must be followed while using geophysical techniques in the health sciences to protect human subjects from radiation exposure, magnetic fields, and other dangers
Biological noise	Background noise produced by biological tissues may conceal signals of interest. Techniques for signal processing must be specifically designed to successfully remove biological noise while maintaining important data
Multi-modality integration	Geophysical approaches must be seamlessly coordinated with other medical imaging modalities and involve the merging of data from many sources. To offer thorough insights, this necessitates complex software and algorithms
Ethical considerations	The use of geophysical methods in the health sciences frequently entails using patient data and human participants. To ensure ethical and accountable research practices, patient consent and data privacy must be taken into account
Biological models	It is crucial to create precise models that accurately depict the intricate behavior of actual tissues. These models help with the interpretation of results in the context of biology and assist the adaption of geophysical approaches
Interdisciplinary collaboration	Effective teamwork amongst professionals from various backgrounds is necessary to close the gap between geophysics and the health sciences. To overcome obstacles and make use of each field’s strengths, effective communication and shared understanding are vital

**Table 9:** Potential future scopes that can be developed to facilitate medical imaging techniques.

Scope	Description
Enhanced Imaging and Analysis	In geophysics-driven medical imaging, ML and AI algorithms can enhance image quality, resolution, and noise reduction. These methods could correct data flaws, improving the accuracy and clarity of photographs
Automated Diagnosis and Detection	To automatically discover patterns linked with different medical disorders in geophysical data, ML algorithms can be taught on large datasets. These algorithms might act as “virtual radiologists,” assisting in the quick and precise diagnosis of diseases
Predictive Analytics	AI algorithms could forecast illness progression, identify potential risk factors, and suggest personalized treatment methods by examining previous geophysical and medical data, adding to proactive healthcare initiatives
Precision Medicine	Geophysical data analysis powered by AI can assist in customizing medical interventions for specific patients. This could entail anticipating treatment outcomes based on tissue characteristics, improving drug distribution, and reducing adverse effects.
Real-Time Monitoring	Geophysical data might be continuously monitored by ML-enabled systems, alerting medical staff to minute changes in tissue qualities. This might come in handy during operations or in critical care settings
Cross-Modality Fusion	Geophysical data and other types of medical imaging can be combined using AI algorithms to create a full picture of a patient’s state. This might result in more precise diagnoses and wiser treatment choices
Automated Workflow Optimization	Geophysical data processing and gathering in medical applications can be streamlined with ML. It might alter the scanner settings in real-time, speeding up scans and improving patient comfort
Novel Biomarkers	Geophysical data analysis powered by AI may produce new biomarkers for tracking the development of disease. These biomarkers may be used to monitor and detect diseases like cancer and neurological illnesses early on
Unveiling Complex Interactions	ML can reveal complex connections between geophysical characteristics and biological functions. This may provide a deeper understanding of the progression of diseases and the impact of treatments on tissue characteristics
Data-Driven Research	By automating data analysis and hypothesis testing, the use of AI in geophysics-driven medical imaging can speed up research while letting scientists concentrate on analyzing data and formulating studies

## Data Availability

The data presented in this review are supported by the inserted references.
